# Multi-Objective Optimization of Curing Profile for Autoclave Processed Composites: Simultaneous Control of Curing Time and Process-Induced Defects

**DOI:** 10.3390/polym14142815

**Published:** 2022-07-10

**Authors:** Wenyuan Tang, Yingjie Xu, Xinyu Hui, Wenchang Zhang

**Affiliations:** 1 State IJR Center of Aerospace Design and Additive Manufacturing, Northwestern Polytechnical University, Xi’an 710072, China; tangwy@mail.nwpu.edu.cn (W.T.); huixinyu@mail.nwpu.edu.cn (X.H.); zhangwc@mail.nwpu.edu.cn (W.Z.); 2 Shaanxi Engineering Laboratory of Aerospace Structure Design and Application, Northwestern Polytechnical University, Xi’an 710072, China

**Keywords:** CFRP, curing process, curing deformation, multi-objective optimization, finite element

## Abstract

The contribution of this work is introducing a multi-objective optimization method based on finite element (FE) numerical simulation to simultaneously control the curing time and cure-induced defects of C-shaped composites during a curing cycle. Thermochemical and thermomechanical coupled analysis is performed and validated experimentally to understand the evolution details of temperature, degree of cure and curing deformation. Aiming to achieve the simultaneous control of manufacturing cost and composite quality, the curing profile is optimized by employing the critical factors including the total curing time, the maximum degree of cure difference, and the curing deformation. The optimization result shows that the designed curing profile can effectively reduce the curing time and guarantee the curing quality. The total curing time of the optimization is reduced by 19%. The verification experiment is also conducted to prove the accuracy and effectiveness of the proposed optimization method.

## 1. Introduction

In recent years, carbon-fiber-reinforced thermoset polymer (CFRP) composite structures have been widely used in aeronautics [1] and aerospace [2], automotive [3], marine [4] and civil engineering [5] owing their high specific stiffness and strength. Autoclave solidification process is one of the most used methods to manufacture the CFRP composites, particularly the primary load-bearing structures in aircraft [6,7,8,9]. In an autoclave process, composites undergo a crosslinking reaction under the prescribed temperature and pressure. The quality of the composites strongly depends on the temperature and pressure curing profiles of the manufacturing process. Unreasonable curing profiles can result in some defects, such as temperature overshoots and non-uniform degree of cure within the composite parts, even the curing deformation [10]. Most of the industry manufacturers adopt the relative conservative curing profiles with long curing time to produce composites. Conventionally, the long curing time negatively affects the manufacturing productivity and leads to a very high energy cost.

Therefore, it is necessary to achieve a more appropriate curing profile which reduces both the process induced defects and curing duration and then improves the high-performance of the composites with low costs. Better understanding of the physical state of composite during crosslinking reaction is essential for developing an optimization method of curing profile. Several experimental contributions have listed. Hui et al. [11] investigated the composite state evolution as temperature, pressure and viscosity during the composite solidification. Almazan-Lazaro et al. [12] observed the impregnation velocity of resin in VARI process by computer vision. Kahali Moghaddam et al. [13] studied the influence of pressure on curing process by embedding different types of pressure sensors in composite. Sorrentino et al. [14] monitored composite temperature evolution using the thermocouples inserted in prepreg sheets. Silva et al. [15] detected the temperature and fluidity of the polymer under compound extrusion manufacturing processes. However, experimental approach is inefficient and the sensor introduced would affect the curing process. Consequently, numerical simulation of the curing process is a relatively efficient and economical approach. Process modeling of polymerizing of thermoset polymer composites have improved from simple one-dimensional finite-difference models [16] to advanced three-dimensional finite element models [17]. Compared with the experimental method in Liu et al. [18], simulation approach could provide comprehensive information during the cure cycle more efficient, such as the trend of temperature [19], degree of cure [20], residual stress of cured part [21] and so on.

Thus, the optimizations of curing profiles based on numerical simulation models of the curing process have become increasingly popular in the literature. For instance, Struzziero and Skordos [22] introduced a multi-objective optimization procedure that combined a finite element solution of process modeling with a genetic algorithm to mitigate the temperature overshoot and cure duration. Axenov et al. [23] proposed a Pareto-based multi-objective optimization method based on curing process simulation to capture optimal two-dwell curing profile for shell-like composite cure quality controlling in autoclave. Rubino et al. [24] studied a fuzzy logic and artificial neural network based computing method to predict the evolution of degree of cure and temperature during curing in autoclave, and optimize the cure profile of the composite. Oh and Lee [25] studied the curing of glass-epoxy composite laminates, and found the optimal value of the curing profile parameter to minimize the overshoot of the laminate. Pantelelis et al. [26] combined genetic algorithm and one-dimensional process model to optimize the optimal curing profile parameters of thermoset matrix composites, thereby shortening the curing duration under the constraints of maximum final degree of cure. Zhang et al. [27] developed a four dwell curing profile for solving the overshoot of degree of cure and temperature during curing process for thick CFRP composite laminates.

However, the present existing work rarely introduces the cure-induced defects into the optimization problem. Motivated by this idea, this paper develops a methodology integrating numerical simulation of curing process with particle swarm optimization algorithm to optimize the curing profile for the C-shaped CFRP composite specimens. The simultaneous control of curing time and process-induced defects is achieved by minimizing the curing time with constraints of the typical defects including non-uniform degree of cure, insufficient cure and curing deformation. Finally, two groups of C-shaped CFRP specimens are produced by the optimal curing profile and the profile recommended by manufacturer. The test and comparison of the parts illustrate well the capability of the developed optimization methodology.

## 2. Materials and Methods

### 2.1. Curing Experiment of C-Shaped Specimens

**Figure 1:**
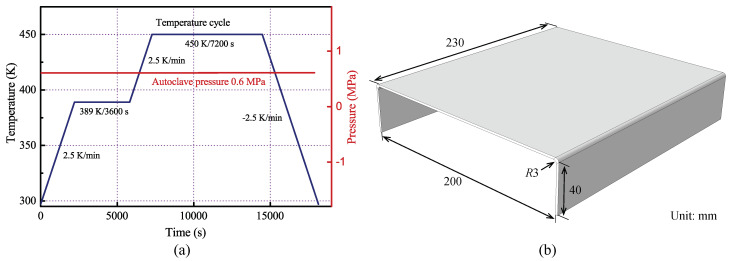
Experimental design of the C-shaped composite specimen: (**a**) Curing profile; (**b**) Geometric size.

**Figure 2:**
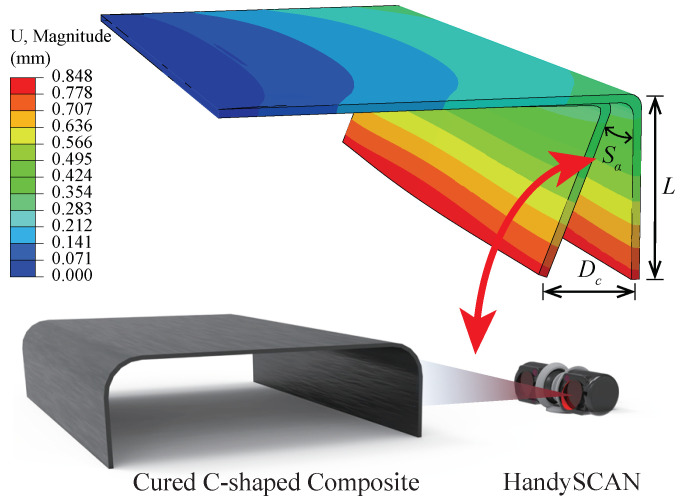
The predicted spring-back angel of the composites with layup [0/90]4S and schematic of laser scanning experiment.

The C-shaped AS4/3501-6 composite specimens were manufactured under the recommended curing profile (see in Figure 1a) and the deformation after curing process was measured to evaluate the composite quality. Three groups of specimens with layup ${\left[0/90\right]}_{4S}$, ${\left[0/45/\;-\;45/90\right]}_{2S}$ and ${\left[45/\;-\;45\right]}_{4S}$ were designed, where 0° direction was the length direction. The size of the each C-shaped specimen was 230 mm in length, 200 mm in web depth and 40 mm in flange width, as illustrated in Figure 1b. To quantify the deformation caused by curing process, the spring-back angle $S_{a}$ was introduced and recorded by the HandySCAN 300 laser scanner, as shown in Figure 2. The average values of the measured specimens with different layups were summarized in Table 1.

### 2.2. Numerical Simulation of Curing Process and Verification

To improve the accuracy of curing process simulation, many physics-chemical based views of composite curing involve numerous interacting systems and a variety of process-dependent parameters that must be accounted for. The thermo-chemical model is established to predict the evolution of degree of cure and temperature of the composites. Then the obtained temperature and degree of cure fields are employed into the thermo-mechanical model. The detailed modeling framework is presented as follow.

#### 2.2.1. Thermo-Chemical Coupled Simulation

The evolution of the degree of cure and temperature during the C-shaped specimen curing process is calculated by a thermos-chemical coupled heat transfer simulation. The thermo-chemical coupled heat transfer equation is expressed as [28]:

(1) $$\rho_{c}C_{c}\frac{\partial T}{\partial t}=K_{x}\frac{\partial^{2}T}{\partial x^{2}}+K_{y}\frac{\partial^{2}T}{\partial y^{2}}+K_{z}\frac{\partial^{2}T}{\partial z^{2}}+\rho_{r}\left(1-V_{f}\right)H_{r}\frac{da}{dt}$$

where $p_{c}$ and $C_{c}$ are the density and specific heat of the composite, respectively. *T* is the current temperature and *t* is the curing reaction time. $K_{i}\;\left(i=x,y,z\right)$ represent the thermal conductivities of the composite. $p_{r}$ is the resin density, $V_{f}$ is the fiber volume fraction, and $H_{r}$ is the total heat energy released from curing reaction of a unit mass of the resin. $\frac{da}{dt}$ is the instantaneous curing rate of the resin and can be determined by the cure kinetics model.

**Figure 3:**
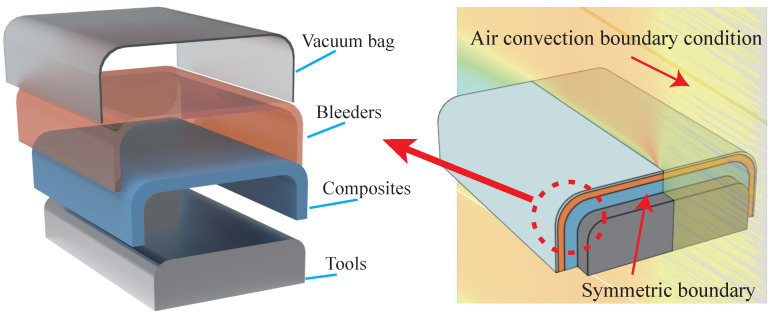
Temperature boundary conditions.

Considering the structural symmetry of the C-shaped composites, 1/2 model is constructed to solve the heat transfer analysis using finite element package ABAQUS. The finite element model meshed with three-dimensional 8-node linear heat transfer brick (DC3D8) elements. The temperature boundary conditions of the model are illustrated Figure 3, where $T_{m}$ is the mold temperature, $T_{g}$ and $P_{g}$ are the air temperature and pressure in the autoclave, respectively. The heating air is used to heat the composites, and the film condition is loaded on the composites surface. The curing profile shown in Figure 2 is imposed on the inner surface of the composites contacting with the mold.

The cure kinetics model of the resin, specific heat capacity and thermal conductivities of the composite are all implemented through the user subroutines UMATHT and USEFLD. The cure kinetics model for the 3501-6 resin is expressed as [29]:

(2) $$\frac{da}{dt}=\left\{\begin{array}{c} \left(K_{1}+K_{2}a\right)\left(1-a\;\right)\left(0.47-a\;\right),\;\;\;\;\;\;a\;\leq 0.3\\ K_{3}\left(1-a\;\right),\;\;\;\;\;\;\;\;\;\;\;\;\;\;\;\;\;\;\;\;\;\;\;\;\;\;\;\;\;\;\;\;\;\;\;\;\;\;\;\;\;\;\;\;\;\;\;\;\;\;\;\;\;\;\;\;\;\;\;\;\;\;\;\;\;\;\;\;\;\;\;\;\;\;\;\;\;\;\;\;\;\;\;\;\;\;\;\;\;\;\;\;\;\;\;a\;>0.3 \end{array}\right.$$

where $k_{i}\left(i=x,y,z\right)$ represent the curing rate constants and can be further defined by the Arrhenius equations:

(3) $$K_{i}=A_{i}\exp\left(\frac{-\Delta E_{i}}{RT}\right),\;\;\;\;\;i=1,\;\;\;\;\;2,\;\;\;\;\;3$$

where $A_{i}$ are the pre-exponential factors. $\Delta E_{i}$ are the reaction activation energies and *R* is the universal gas constant.

The specific heat capacity of the composite *C* is calculated using the rule of mixture [30,31]:

(4) $$C=w_{f}C_{f}+\left(1-w_{f}\right)C_{r}$$

where $w_{f}$ is the weight fraction of the fiber. $C_{f}$ and $C_{r}$ denote the specific heat capacity of fiber and resin, respectively.

(5) $$w_{f}=\frac{V_{f}P_{f}}{V_{f}P_{f}+\left(1-V_{f}\right)P_{r}}$$

The longitudinal ($K_{x}$) and transverse direction ($K_{y}$ and $K_{z}$) thermal conductivities of the composite are computed as [32]:

(6) $$K_{x}=V_{f}K_{f}^{x}+\left(1-V_{f}\right)K_{r}$$

(7) $$K_{y}=K_{z}=V_{f}K_{r}\left(\frac{K_{f}^{y}}{K_{r}}-1\right)+K_{r}\left(\frac{1}{2}-\frac{K_{f}^{y}}{2K_{r}}\right)+K_{r}\left(\frac{K_{f}^{y}}{K_{r}}\;-\;1\right)\left(V_{f}^{2}-V_{f}+\frac{{\left(\frac{K_{f}^{y}}{K_{r}}\;+\;1\right)}^{2}}{4{\left(\frac{K_{f}^{y}}{K_{r}}-1\right)}^{2}}\right)$$

where $K_{f}^{x}$ and $K_{f}^{y}$ are the longitudinal and transverse thermal conductivities of the fiber. $K_{r}$ is the thermal conductivity of the resin. Considering the model in this study is macro scale and the size of the C-shaped specimens is small, the influence of Kapitza resistance [33,34] on the heat transfer of air-composite interface (and also at the fiber-resin interface) is ignored in the simulation process.

The parameters values for Equations (1)–(7) are reported in Table 2. Note that the specific heat capacity and thermal conductivity of AS4 fiber has a linear dependence on temperature, while the specific heat capacity and thermal conductivity of the resin depends on both the degree of cure and temperature.

#### 2.2.2. Thermo-Mechanical Coupled Simulation

The calculated degree of cure and temperature are further used to predict the residual stress and strain in the cured composites. It has been demonstrated that the thermoset resin composite exhibits viscoelastic behavior during curing process [36]. The strain tensor of the composite ε in the curing process can be described as [37]:

(8) $$\varepsilon =\varepsilon^{e}+\varepsilon^{th}+\varepsilon^{ch}$$

where $\varepsilon^{e}$ and $\varepsilon^{th}$ represent the viscoelastic and thermal expansion strain, which are determined by the viscoelastic and thermal expansion properties of the composite. $\varepsilon^{ch}$ is the chemical shrinkage strain of the composite and can be calculated by

(9) $$\varepsilon_{x}^{ch}=\delta_{x}\Delta a$$

(10) $$\varepsilon_{y}^{ch}=\delta_{y}\Delta a$$

where $\varepsilon_{x}^{ch}$ and $\varepsilon_{y}^{ch}$ represent the longitudinal and transverse chemical shrinkage strains of the composite, respectively. $\delta_{x}$ and $\delta_{y}$ are the longitudinal and transverse chemical shrinkage coefficients of the composite.

**Figure 4:**
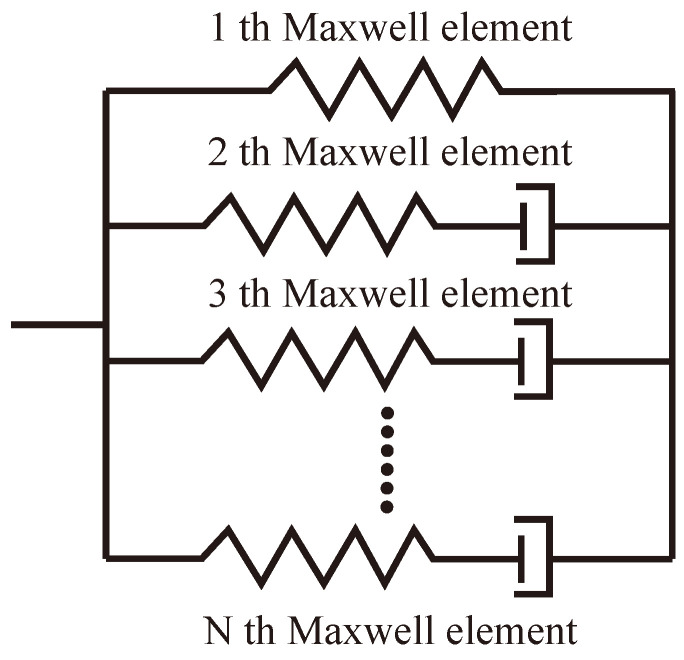
Illustration of the generalized Maxwell model.

In this study, a linear viscoelastic constitutive equation [38] is used to model the composite:

(11) $$\sigma \left(t\right)=\int_{0}^{t}C\frac{\partial \varepsilon^{e}}{\partial \tau }d\tau$$

where σ represents the stress tensor of composite. *t* and τ denote the current time and past time, respectively. C is the stiffness matrix of composite and can be modeled by generalized Maxwell equation. The stiffness modeled by a parallel connection of *n* spring-dashpot elements, as shown in Figure 4, can be further approximated by the Prony series as:

(12) $$C=C_{\infty }+\left(C_{u}-C_{\infty }\right)\sum_{i=1}^{n-1}w_{i}\exp\left(\frac{-t}{\tau_{i}\left(a\;\right)}\right)$$

where $C_{\infty }$ is the stiffness matrix of the fully relaxed (rubbery) composite and $C_{u}$ is the stiffness matrix of the unrelaxed (glassy) composite. $w_{i}$ and $\tau_{i}\left(a\;\right)$ denote the weight factor and the relaxation time for the *i*th Maxwell element, respectively.

It is assumed that the $C_{\infty }$ and $C_{u}$ of AS4/3501-6 composite are constants independent of temperature and degree of cure. The relation between $C_{\infty }$ and $C_{u}$ are described as:

(13) $$C_{\infty }=rC_{u}$$

where *r* is a constant between 0 and 1. For the AS4/3501-6 composite, the value of *r* is 0.14 [36]. The unrelaxed materials properties for AS4/3501-6 composite are listed in Table 3. The stiffness matrix coefficients of $C_{u}$ can be derived from the unrelaxed modulus and Poisson’s ratio.

The relaxation time $\tau_{i}\left(a\right)$ is the function of the degree of cure and can be expressed as:

(14) $$\tau_{i}\left(a\right)=10^{\log\left(\tau_{i}\left(a_{c}\right)\right)+\left(f\left(a\right)-\left(a-a_{c}\right)\right)\log\left(\theta_{i}\right)}$$

where $\tau_{i}\left(a_{c}\right)$ is the relaxation time under reference degree of cure. f(a) and $\theta_{i}$ are formulated as [40]:

(15) $$f\left(a\right)=9.137a^{2}+0.6089a-9.3694$$

(16) $$\theta_{i}=\frac{10^{9.9}}{\tau_{i}\left(a_{c}\right)}$$

The weight factor $w_{i}$ and the relaxation time at reference degree of cure $\tau_{i}\left(a_{c}\right)$ for AS4/3501-6 are listed in Table 4.

**Figure 5:**
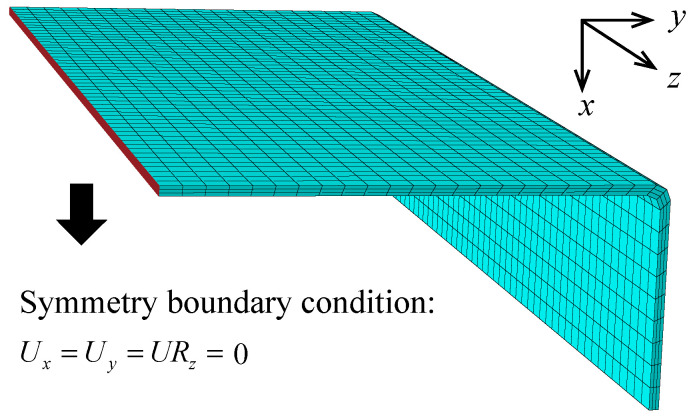
The established finite element model.

The thermo-mechanical coupled simulation is conducted within ABAQUS. User subroutines UMAT and UEXPAN are used to model the constitutive behavior of the composites. A static implicit analysis is used to calculate the stresses and strains during the curing process. Then, another analysis step is conducted to calculate the curing deformation induced by the curing residual stresses. The displacement boundary condition in the static analysis is shown in Figure 5, where the axial movement of *x* and *y* and the rotation of the *z* axis are constrained.

#### 2.2.3. Verification for Simulation

**Figure 6:**
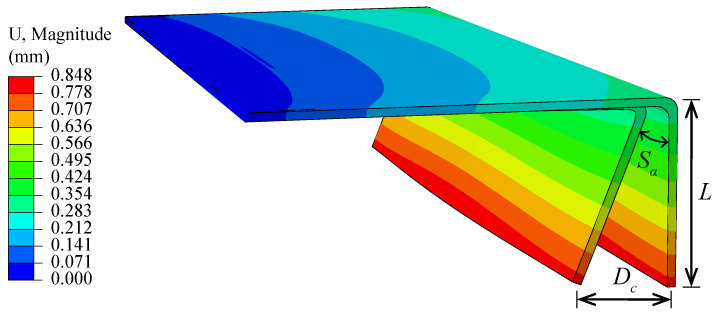
The predicted curing deformation of the composites with layup ${\left[0/90\right]}_{4S}$.

By using the thermal-chemical and thermal-mechanical analysis, the cure-induced deformations of the C-shaped composites are obtained. Figure 6 presents the deformation of the composite with layup ${\left[0/90\right]}_{4S}$. The predicted spring-back angle $S_{a}$ of the composite can be defined by:

(17) $$S_{a}=\arctan\left(D_{c}/L\right)$$

where $D_{c}$ is the maximum displacement of the composite after curing process and *L* is the length of the composite flange.

**Figure 7:**
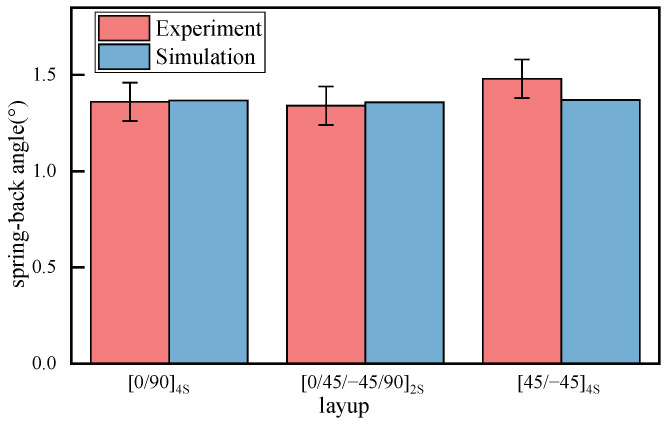
The predicted curing deformation of the composites with layup ${\left[0/90\right]}_{4S}$.

To validate the accuracy of curing simulation, the calculated spring-back angle of the model is compared with the experiments, as summarized in Figure 7. The comparison shows good agreement with the composites of different layups, which highlights the prediction capacity of the proposed models.

**Figure 8:**
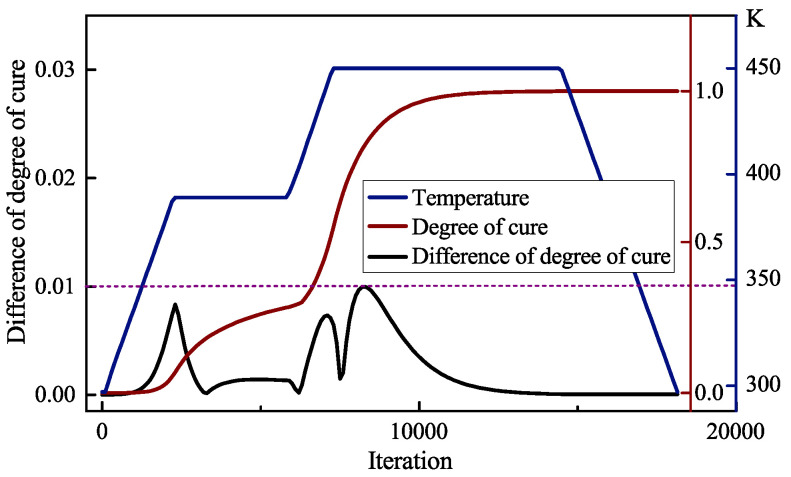
Evolution history of maximum difference of degree of cure.

In order to further evaluate the overall curing quality of the composite, the evolutions of the minimum degree of cure of the whole model are extracted. The minimum value exceeds 0.95 at the end of the curing process, which indicates that the composite part has completed cure reaction. In order to avoid residual stress caused by degree of cure distributed unevenly in the composites, the simultaneous difference of degree of cure $a_{d}$ is estimated from the center and bottom of the model, as shown in Figure 8. The first peak of $a_{d}$ appears after the first heating stage, since the first degree of cure rises rapidly. Then $a_{d}$ keep holding for a while and tends to reduce owing the thin-walled composites are easy to achieve isothermal. For the second heating stage and the early stage of second dwell stage, the curing reaction becomes severe, which induces the temperature difference between the middle and outer regions of the composite part. A transient relief appears when the degree of cure of surface is exceeded by center. With the end of the curing reaction, $a_{d}$ is reduced gradually and finally remains stable. The global maximum difference of degree of cure at the end of the curing process is 0.01.

### 2.3. Optimization of Curing Profile

#### 2.3.1. Optimization Problem Formulation

**Figure 9:**
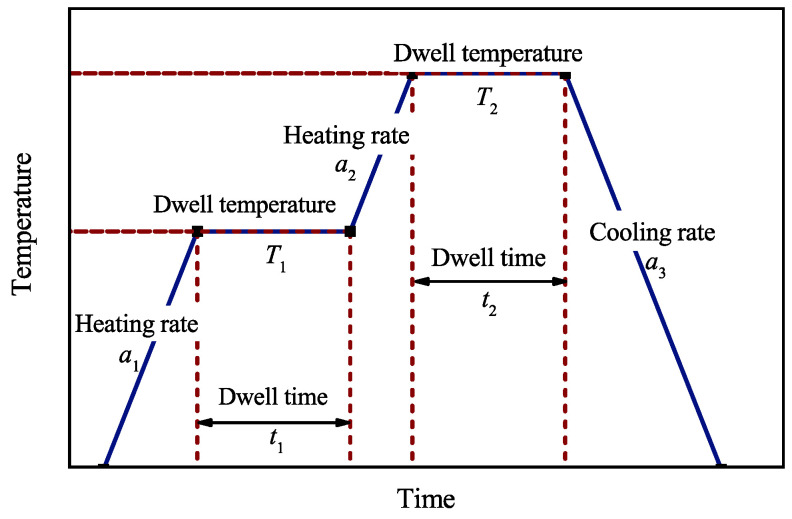
Two-dwell curing profile.

Based on the curing simulation result, an optimization problem is established for simultaneous control of curing time and process-induced quality of the composites. C-shaped AS4/3501-6 with ${\left[0/45/\;-\;45/90\right]}_{2S}$ layup is used as the optimization model. Two-dwell curing profile as shown in Figure 9 is optimized to shorten the curing time and the typical defects (non-uniform degree of cure and spring-back angel) with maintaining sufficient cure level of the composites. Six parameters including the two heating rate ($a_{1}$, $a_{2}$), two dwell time ($t_{1}$, $t_{2}$), and two dwell temperature ($T_{1}$, $T_{2}$) are extracted from the curing profile as the design variables.

The optimization problem can be formulated mathematically as:

(18) $$\begin{array}{c} Minimize\;\;\;\left\{\begin{array}{c} t_{total}\;\;\;\left(a_{1},\;\;\;\;a_{2},a_{3},\;\;\;\;T_{1},\;\;\;\;T_{2},\;\;\;\;t_{1},\;\;\;\;t_{2}\right)\\ a_{d}\;\;\left(a_{1},\;\;\;\;a_{2},a_{3},\;\;\;\;T_{1},\;\;\;\;T_{2},\;\;\;\;t_{1},\;\;\;\;t_{2}\right)\\ S_{a}\;\;\left(a_{1},\;\;\;\;a_{2},\;\;a_{3}\;,\;\;T_{1},\;\;\;\;T_{2},\;\;\;\;t_{1},\;\;\;\;t_{2}\right) \end{array}\right.\;\;\;\;\;\;\;\;\;\;\\ Subjected\;\;\;\;to\;\;\;\;a_{f}\;\;\left(a_{1},\;\;\;\;a_{2},\;\;\;a_{3},T_{1},\;\;\;\;T_{2},\;\;\;\;t_{1},\;\;\;\;t_{2}\right)\geq 0.95 \end{array}$$

where $t_{total}$ is the total curing time. $a_{d}$ is the maximum difference of degree of cure during the curing process. $a_{f}$ is the minimum degree of cure in the composite specimen after cure and $S_{a}$ is spring-back angel of the composites after cure. To solve the optimization problem, the multi-objective particle swarm optimization (MOPSO) algorithm is introduced to yield a global optimum from the cure simulation model.

#### 2.3.2. Particle Swarm Optimization Algorithm

The MOPSO algorithm can handle both linear and nonlinear optimization problems [41]. In MOPSO algorithm, every particle is designed to simulate the behavior of birds that fly in the design space. Their velocity and position not only could be affected by the best position that the particles flied, but also the position saved in archive. Under the effect of particle swarm and archive swarm, the MOPSO particle swarm algorithm retains diversity, besides demonstrates its search capabilities. According the good position, the particles update their position and fly velocity based on update formula as follows:

(19) $$V_{i}^{k+1}=\omega V_{i}^{k}+c_{1}r_{1}\left(P_{i}^{k}-X_{i}^{k}\right)+c_{2}r_{2}\left(ARC\left[j\right]-X_{i}^{k}\right)$$

(20) $$X_{i}^{k+1}=X_{i}^{k}+V_{i}^{k+1}$$

here $X_{i}$ and $V_{i}$ represent position coordinate and velocity coordinate of the *i*th individual particle, respectively, (the subscripts *k* and k+1 refer to the current and the next iterations, respectively). The first part of the formula used for updating the velocity describes the influence of the previous velocity on the current velocity. Inertia weight as proxies to quantification the influence, and is set to 0.875 in this study. The second part of the formula relies on the distance between the recent position and the best position of the particle that has flown so far. $P_{i}^{k}$ is the best previous position of the *i*th particle. $c_{1}$ is stochastic acceleration terms as proxies to the acceleration factor that attracts each particle towards the good position in its own experience. The third part depends on the distance between the recent position and the chosen particle from the archive. ARC[j] is a particle position taken from the archive. The index *j* is selected in the following way: After collecting the new position respecting the non-dominated solution, the adaptive gird the particle points in the archive can be wrapped by the hypercube, and a fitness value is assigned to estimate the density of the hypercube, which is inversely proportional to congestion. If the archive is full, the hypercube with the lowest fitness value will delete one particle to release storage for the new particle. After estimation, roulette-wheel selection is used to choose the hypercube based on the fitness value, and *j* is randomly selected within such hypercube. The size of archive is 20. $c_{2}$ is stochastic acceleration terms as proxies to the acceleration factor which attract each particles towards the chosen particle from the archive. In this work, $c_{1}$ = 2 and $c_{2}$ = 2 are set. $r_{1}$ and $r_{2}$ are two random constants from [0,1].

The problem-specific constraints and the layout of the variable are included in most optimization problems. For the optimization in this study, the problem-specific constraints are the minimum final degree of cure. The layout of the variable is design bounds of the curing profile parameters, where the allowable ranges are listed in Table 5. The solution would be ignored, if the particle searches out of the layout of the variable, even the problem-specific constraint is satisfied. So making sure none of the particles reach the outside layout of the variable and verifying whether met the problem-specific constraint is crucial. In this paper, the particles reach outside range is handled by the harmony search algorithm-based method. The detailed descriptions of the harmony search algorithm-based method can be found in previous studies [42]. The MOPSO algorithm is integrated with the curing simulation model and the Pseudo code of optimization is shown in Algorithm 1. For the present optimization problem, a population of 20 particles is used, the maximum number of generations is set as 50, and the maximum velocity is 5.

**Algorithm 1:** Pseudo code of optimization.  Initialize particles to random position and velocities to zero
  **Repeat**
    Update global best and particles best
    Populate archive with the positions that represent nondominated solation
    Regenerate adaptive grid
      **For** each particle **do**
 
      compute particles velocity according to
      Equation (19) above
      calculate particles position according to
      Equation (20) above
      Perform cure simulation model
      evaluate particle fitness
    **End Do**
  **Until** Termination condition met


## 3. Results and Discussion

**Figure 10:**
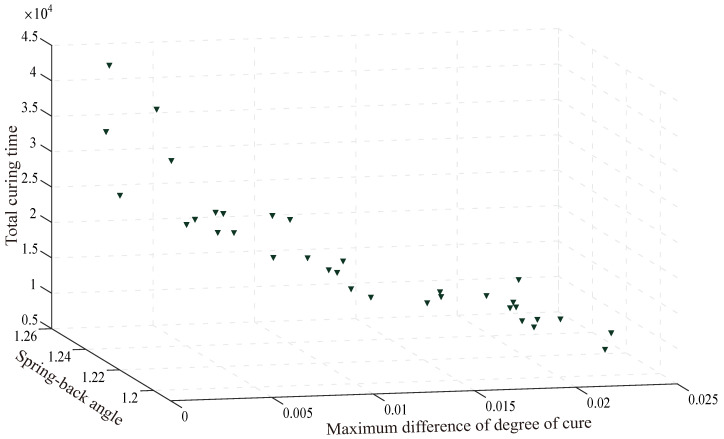
Three-dimensional scatter plot of the Pareto front of the multi-objective optimization result.

**Figure 11:**
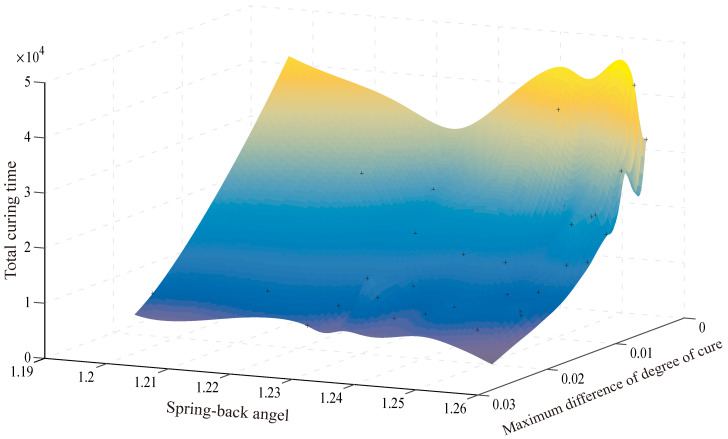
Surface plot of the Pareto front of the multi-objective optimization result.

The optimization process is executed using a twelve-core processor (2.8 GHz) and 50 GB memory. The optimization converges after 700 iterations and takes about 50 h. The multi-objective optimization results are illustrated in Figure 10 and Figure 11. As can be seen, the 3D contour plot of the optimal Pareto front suggests the three objectives ($a_{d}$ difference of degree of cure, $S_{a}$ spring-back angel and $t_{total}$ total curing time) have the following relationship: If the differences of degree of cure and the spring-back angel are prioritized, the curing time will be prolonged. If focusing on the total curing time, differences of degree of cure and spring-back angel will be unsatisfactory. In order to achieve simultaneous control of curing time and process-induced defects, it is necessary to select the most reasonable curing profile among the number of Pareto fronts according to balancing the weight of total curing time, the maximum differences of degree of cure and spring-back angle. Therefore under the premise of ensuring the curing quality, the result of minimizing the curing time is selected.

**Figure 12:**
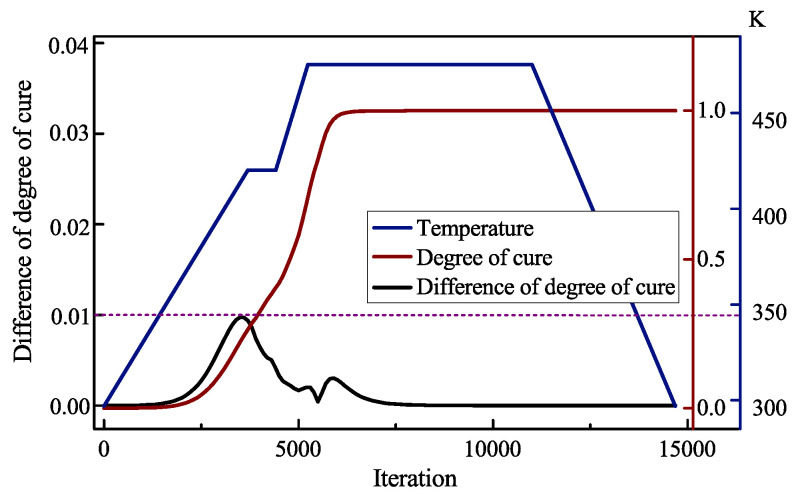
Maximum difference of degree of cure evolution.

The optimized curing profile selected from Pareto front are reported in Table 6. For comparison, the parameters of the initial curing profile are also included. It is found that the optimized curing profile reduces to 14,686 s by about 19%. Figure 12 presents the optimized curing profile. It is observed that the first heating rate decreases from 2.5 K/min to 2.0 K/min and the first dwell temperature rises to 420.1 K. Properly increasing the dwell temperature helps to advance the curing reaction, and reducing the heating rate can effectively avoid the aggravation of the temperature difference caused by the curing exothermic. Besides, it is considered that the resin exhibits a viscous or highly elastic state in the early stage when the degree of cure is low. The premature peak of degree of cure difference weakens the cure-induced residual stress and further deformation. The first dwell time decreases from 3600 s to 720 s. To improve the cure efficiency, the first dwell time should not be long, because the temperature at this time is not sufficient to completely cure the composites. After the first peak, there has been an equilibrium period and the optimal curing temperature has not yet been reached. If it enters the second heating stage at this time, the curing cycle can be effectively shortened. After optimized, the second heating rate increase from 2.5 K/min to 4.0 K/min. In the second heating stage, higher heating rate push the temperature to reach the required temperature faster, which can accelerate curing reactive at heating stage. A higher heating rate causes the surface temperature of the composites to rise rapidly, which thereby reduces the temperature overshoot caused by internal curing exothermic and keeps the temperature and curing rate of the center and surface of the composites at a relatively same level. The second dwell temperature increase from 450 K to 475.2 K and the dwell time rises from 3600 s to 5760 s. The higher dwell temperature promotes the curing rate and the longer dwell time makes the reaction complete in the second heating stage to avoid large temperature and degree of cure difference. Although the spring-back deformation of the part can be reduced by reducing the cooling rate, the change from 2.5 K/min to 2.9 K/min has little effect on the deformation but can effectively reduce the curing time.

**Figure 13:**
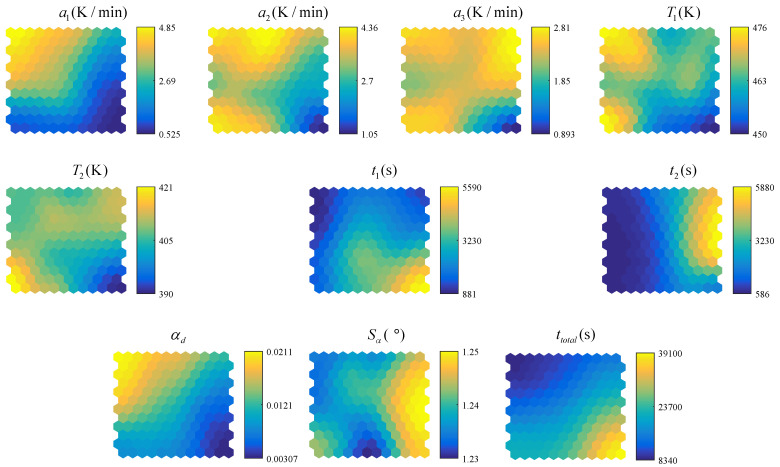
Maximum difference of degree of cure evolution.

The self-organizing map (SOM) can provide visualization between design parameters and optimization objectives and inspire designers how design variables affect optimization objectives [43]. Figure 13 describes the relationships between the design variables and objectives with respect to the curing process using SOM. The label of each map represented by color indicates the weight value of the design variable of the optimization network, where the blue denotes the lower limit and the yellow denotes the higher limit. The relationship between the design parameters and objectives can be found by comparing the distribution law of the color map in a visualized approach.

It is observed in Figure 13 the distribution of the first heating rate $a_{1}$ is similar to degree of cure difference $a_{d}$, but opposite to total curing time $t_{total}$, which suggests a higher $a_{1}$ make the $a_{d}$ increase and leads to a shorter $t_{total}$. Based on the color map, the influences of other variables can be derived by the same way. For instance, the $a_{d}$ can be reduced by reducing the heating rate of the two stages $a_{1}$ and $a_{2}$, increasing the dwell time of the first stage $t_{1}$, or reducing the dwell temperature of the two stages $T_{1}$ and $T_{2}$.

### Experimental Verification

**Figure 14:**
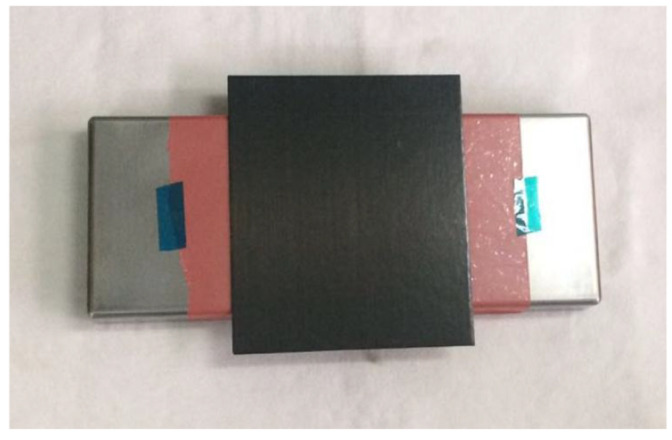
Prepregs and mold.

In order to verify the proposed multi-objective optimization method, the initial and optimized curing profiles shown in Table 6 are both implemented for the AS4/3501-6 C-shaped composites using autoclave system. The geometric size and layup property of the composites are remain consistent. Figure 14 illustrates the prepregs and the mold before entering the autoclave. After curing process, the average values of the spring-back angle are measured and compared with the numerical results, as shown in Table 7.

As far as the manufacturing cost and efficiency is concerned, the curing time of the optimized profile is shortened by 19% compared with the initial profile, which indicates the feasibility of the proposed optimization method.

## 4. Conclusions

A multi-objective optimization method is proposed to design the two-dwell curing profile of the CFRP composites to simultaneously control curing time and cure-induced defects. Through the thermo-chemical and thermo-mechanical coupled analysis, the evolution history of uneven cure and spring-back deformation is firstly evaluated and validated by the experiments. Then the curing profile which controls the composite manufacturing is optimized to improve the cure quality and costs through the multi-objective particle swarm algorithm. The verification experiment is also conducted to prove the accuracy and effectiveness of the optimization method. The result indicates that using the optimized curing profile achieves a better standard including high degree of cure, short curing time, and reliable quality controlled. Compared with the initial profile, the total curing time is shortened to 14,686 s, which is reduced by 19%. The self-organizing map further visualizes the relationship between the design parameters of the curing profile.

## Figures and Tables

**Table 1:** The measurement value of the spring-back angle.

Layup	Experiment (${}^{\circ }$)
${\left[0/90\right]}_{4S}$	1.34
${\left[0/45/\;-\;45/90\right]}_{2S}$	1.36
${\left[45/\;-\;45\right]}_{4S}$	1.48

**Table 2:** Parameters values for the cure kinetics, specific heat capacity and thermal conductivities of resin [35].

Material	Property	Value
3501-6 resin	$p_{r}$ $(\mathrm{kg}/m^{3})$	1272
	$C_{r}$ (J/(kg·K))	$4184\times (0.468+5.975\times 10^{-4}\times T-0.141a)$
	$K_{r}$ (W/(K·m))	0.04184×(3.85+(0.035×T−0.141)a)
	$H_{r}$(J/kg)	473,600
	*R* (J/(mol·K))	8.3143
	$A_{1}$ $\left(\min^{-1}\right)$	$2.101\times 10^{9}$
	$A_{2}$ $(\min^{-1})$	$-2.014\times 10^{9}$
	$A_{3}$ $(\min^{-1})$	$1.96\times 10^{5}$
	$\Delta E_{1}$ (J/mol)	$8.07\times 10^{4}$
	$\Delta E_{2}$ (J/mol)	$7.78\times 10^{4}$
	$\Delta E_{3}$ (J/mol)	$5.66\times 10^{4}$
AS4 fiber	$p_{r}$ $(\mathrm{kg}/m^{3})$	1790
	$C_{f}$ (J/(kg·K))	750+2.05×T
	$K_{f}^{x}$ (W/(K·m))	7.69+0.0156×T
	$K_{f}^{y}$ (W/(K·m))	2.4+0.00507×T

**Table 3:** Unrelaxed properties of AS4/3501-6 composite [39].

Property	Value
$E_{x}$ (GPa)	126
$E_{y}$ (GPa)	8.3
$G_{xy}$ (GPa)	4.1
$G_{yz}$ (GPa)	2.8
$v_{xy}$	0.3
$v_{yz}$	0.5
$\beta_{x}$ $\left(K^{-1}\right)$	0.5
$\beta_{y}$ $\left(K^{-1}\right)$	35.3
$\delta_{x}$	−0.017%
$\delta_{y}$	−0.88%

**Table 4:** Relaxation times and weight factors at the reference degree of cure ($a_{c}$ = 0.98) [40].

*i*	$\tau_{i}$ (min)	$w_{i}$
i=1	$2.922\times 10^{1}$	0.0569
i=2	$2.922\times 10^{3}$	0.066
i=3	$1.82\times 10^{5}$	0.083
i=4	$1.10\times 10^{7}$	0.112
i=5	$2.83\times 10^{8}$	0.154
i=6	$7.94\times 10^{9}$	0.262
i=7	$1.95\times 10^{11}$	0.184
i=8	$3.32\times 10^{12}$	0.049
i=9	$4.92\times 10^{14}$	0.025

**Table 5:** Range of the design variable.

Design Variable	Parameter	Range	Minimum Increment
First heating rate	$a_{1}$ (K/min)	[0.5, 5.0]	0.1
Second heating rate	$a_{2}$ (K/min)	[0.5, 5.0]	0.1
First dwell temperature	$T_{1}$ (K)	[388, 428]	0.1
Second dwell temperature	$T_{2}$ (K)	[448, 488]	0.1
First dwell time	$t_{1}$ (s)	[600, 6000]	60
Second dwell time	$t_{2}$ (s)	[600, 9000]	60
Cooling rate	$a_{3}$ (K/min)	[0.5, 3.0]	0.1

**Table 6:** Parameters of initial cure profile and optimized curing profile.

Parameters	Initial	Optimized
$a_{1}$ (K/min)	2.5	2.0
$a_{2}$ (K/min)	2.5	4.0
$T_{1}$ (K)	389	420.1
$T_{2}$ (K)	450	475.2
$t_{1}$ (s)	3600	720
$t_{2}$ (s)	3600	5760
$a_{3}$ (K/min)	2.5	2.9
$S_{a}$ (${}^{\circ }$)	1.218	1.256
$t_{total}$ (s)	18,144	14,686
$a_{f}$	1	1
$a_{d}$	0.01038	0.01006

**Table 7:** Comparisons of measurement and simulation results of the spring-back angle.

	Measurement (${}^{\circ }$)	Simulation Result (${}^{\circ }$)
Initial	1.218	1.225
Optimized	1.21	1.18

## Data Availability

The raw/processed data required to reproduce these findings cannot be shared publically but is available upon request.
